# A Pooled Genome-Wide Association Study of Asperger Syndrome

**DOI:** 10.1371/journal.pone.0131202

**Published:** 2015-07-15

**Authors:** Varun Warrier, Bhismadev Chakrabarti, Laura Murphy, Allen Chan, Ian Craig, Uma Mallya, Silvia Lakatošová, Karola Rehnstrom, Leena Peltonen, Sally Wheelwright, Carrie Allison, Simon E. Fisher, Simon Baron-Cohen

**Affiliations:** 1 Autism Research Centre, Department of Psychiatry, University of Cambridge, Cambridge, United Kingdom; 2 School of Psychology and Clinical Language Sciences, Centre for Integrative Neuroscience and Neurodynamics, University of Reading, Reading, United Kingdom; 3 MRC Centre for Social, Genetic and Developmental Psychiatry, King’s College London, Institute of Psychiatry, London, United Kingdom; 4 The Wellcome Trust Sanger Institute, Hinxton, Cambridgeshire, United Kingdom; 5 Max Planck Institute for Psycholinguistics, 6500 AH, Nijmegen, The Netherlands; 6 Donders Institute for Brain, Cognition and Behaviour, Radboud University Nijmegen, Nijmegen, The Netherlands; 7 CLASS Clinic, Cambridgeshire and Peterborough NHS Foundation Trust (CPFT), Cambridge, United Kingdom; Democritus University of Thrace, GREECE

## Abstract

Asperger Syndrome (AS) is a neurodevelopmental condition characterized by impairments in social interaction and communication, alongside the presence of unusually repetitive, restricted interests and stereotyped behaviour. Individuals with AS have no delay in cognitive and language development. It is a subset of Autism Spectrum Conditions (ASC), which are highly heritable and has a population prevalence of approximately 1%. Few studies have investigated the genetic basis of AS. To address this gap in the literature, we performed a genome-wide pooled DNA association study to identify candidate loci in 612 individuals (294 cases and 318 controls) of Caucasian ancestry, using the Affymetrix GeneChip Human Mapping version 6.0 array. We identified 11 SNPs that had a p-value below 1x10^-5^. These SNPs were independently genotyped in the same sample. Three of the SNPs (rs1268055, rs7785891 and rs2782448) were nominally significant, though none remained significant after Bonferroni correction. Two of our top three SNPs (rs7785891 and rs2782448) lie in loci previously implicated in ASC. However, investigation of the three SNPs in the ASC genome-wide association dataset from the Psychiatric Genomics Consortium indicated that these three SNPs were not significantly associated with ASC. The effect sizes of the variants were modest, indicating that our study was not sufficiently powered to identify causal variants with precision.

## Introduction

Asperger Syndrome (AS) is a neurodevelopmental condition and a subset of Autism Spectrum Conditions (ASC) [1]. Individuals with ASC have difficulties in social interaction and communication, alongside unusually repetitive and stereotyped behaviour and unusually narrow interests. In AS, language and cognitive development proceed on time. ASC is highly heritable [2], with monozygotic twin heritability rate estimated between 73 – 95% [3], and has a prevalence of approximately 1% [4]. ASC is characterized by high clinical and aetiological heterogeneity. Environmental, epigenetic and genetic factors have been implicated in ASC [3, 5–7]. Currently, more than 660 genes are implicated in ASC (https://gene.sfari.org/autdb/HG_Home.do), though no single gene or variant accounts for more than 1–2% of cases [5,8]. Additionally, several large copy number variants that duplicate or delete multiple genes have also been identified in association with ASC [3,9].

Though no common variant has been consistently associated with ASC across multiple Genome-wide Association Studies (GWAS) [3,5], it is clear now that they contribute considerably to the variation in ASC [10, 11]. Two recent studies have identified that common inherited variation contributes to between 40 – 60% of the variance in ASC [10, 11]. However, despite the majority of variance attributable to common inherited variants, as explained earlier, genome-wide association studies have failed to consistently identify causative variations. One explanation for this lack of success is that genome-wide association studies in ASC may be underpowered to detect small effect sizes; the largest ASC GWAS had less than 9000 participants (cases and controls) and, although this seems large, it has been argued that much larger sample sizes are needed (in the range of tens and hundreds of thousands) to successfully identify causative variants [9]. An alternative view is that the inability to consistently identify causative common variants is due to the underlying genetic and phenotypic heterogeneity. At a phenotypic level, delay and difficulties in language development is an important source of heterogeneity in ASC. Language delay in individuals with ASC is associated in changes in brain volume in both total grey matter and in specific regions in the brain [12]. This different brain architecture points to different biological and genetic networks involved in different forms of ASC. As mentioned earlier, AS is a subset of ASC where individuals have no language delay, suggesting it may have a genetic architecture distinct from the rest of ASC.

Only a few studies have specifically investigated the genetics of AS. In one of the first such studies, we tested for associations between 216 SNPs across 68 candidate genes. We identified nominal associations between SNPs in 14 genes and AS [13]. In the current study, we performed a pooled DNA genome wide association in individuals with AS and controls to identify SNPs in a hypothesis-free way. DNA pooling is a rapid, efficient and economical method to identify genetic associations in various conditions [14]. We hypothesized that genome-wide DNA pooling would detect the differences in allele frequencies between individuals with AS and controls. SNPs whose p-values were below a pre-defined threshold were then individually genotyped in the same sample using an established approach reported in several previous studies [15–17].

## Methods

### Participants

612 individuals were genotyped in the pooled genotyping stage. There were 294 cases (males = 254, females = 40, reflecting the male bias in AS [5]) and 318 controls (males = 250, females = 68). 607 of these individuals were individually genotyped. 5 (1 case and 4 controls) individuals were not genotyped at this stage due to poor DNA quality. All participants reported Caucasian ancestry for at least 2 generations. All cases were recruited from the Cambridge Autism Research Database (CARD) at www.autismresearchcentre.com, and reported that they had a clinical diagnosis of AS according to DSM IV or ICD-10 criteria. Clinical diagnostic assessment was done by independent clinicians. Control participants were recruited through advertisement and reported that they were free of psychiatric and neurological conditions. Written consent was obtained from all participants. Ethical approval was obtained from the National Health Service Research Ethics Service (NRES).

### Pooled DNA Genotyping

DNA from each participant was extracted from buccal swabs and anonymized. DNA was then suspended in Tris-EDTA and quantified using PicoGreen double-stranded DNA quantification reagent (Invitrogen, USA). 100 ng of DNA from each individual was added to their respective pool. The cases were divided into 7 pools with 5 pools for males and 2 pools for females. On average, there were 42 participants in each pool, though the numbers ranged from 12 to 59. Two additional pools with 24 female cases and 44 male cases were genotyped, but were not included in the analysis or taken forward for individual genotyping due to DNA contamination. The controls were divided into 9 pools with an average of 35 participants per pool. The number of participants per control pool ranged from 14 participants to 57.

Genotyping was performed using the Affymetrix GeneChip Human Mapping version 6.0 array (Affymetrix, California, USA) according to the protocol recommended by the company. Cell intensity (.cel) files were generated using GeneChip Scanner 3000 7g. The files generated were converted into relative allele signal scores (RAS) using a custom made script (snpmap.R [18]).

To test for differences in allele frequencies for each SNP between the cases and the controls, independent t-tests (equal variance assumed) were performed using the mean RAS scores from the pools. In addition, Levene’s test was performed to check for equality of variance. A threshold of significance was chosen *a priori* at p = 1 x 10^−5^. This particular threshold was chosen in order to reduce the risk of false negatives due to the loss of power from DNA pooling [19*] and this is a threshold that is typically used in the discovery phase of GWAS studies. All SNPs were screened for quality control. The study design had approximately 38% power to detect variants with an effect of 1.3 for the given threshold of significance after taking power loss due to DNA pooling into consideration. The frequency of both the marker and the effect allele was 0.5 for the power calculation. SNPs were rejected if they had a minor allele frequency (MAF) below 0.01 in the Caucasian population according to the HapMap project, and if the coefficient of variation (calculated as SD/mean) in more than 50% of the pool was greater than 0.2. All SNPs which passed quality control and had a p-value below the threshold of significance were taken forward for individual genotyping to verify the result from the pooled association. Nominally significant SNPs in the individual genotyping stage were further investigated using summary genome-wide association data of the ASC cohort available from the Psychiatric Genomics Consortium (PGC, http://www.med.unc.edu/pgc/). The PGC analysed genome-wide SNPs using DNA from 161 cases, 526 controls, 4788 trio cases and 4788 trio pseudocontrols, all of Caucasian ancestry. A crucial difference in the PGC cohort from our study cohort is that the PGC cohort did not stratify for language delay (and hence includes cases of autistic disorder/childhood autism as well as AS). Additional details of methods, statistical analyses and participant ancestry are provided elsewhere [20*].

### Individual Genotyping and functional annotation

Individual genotyping was performed by Geneservices UK Ltd using the Sequenom MassARRAY iPLEX platform (Sequenom, San Diego, USA). 5 (1 case and 4 controls) individuals who were genotyped at the pooled DNA analysis stage were not included in this stage due to poor DNA quality. Total genotyping rate was 97%. MAF in the genotyped sample for all the SNPs was above 0.05. Allelic association testing was performed using Plink v1.07 (http://pngu.mgh.harvard.edu/~purcell/plink/) [21]. Functional annotation was performed using Haploreg v2 (http://www.broadinstitute.org/mammals/haploreg/) [22] and SNPnexus (http://www.snp-nexus.org/) [23].

### Validation of DNA pooling and replication

To assess the accuracy of DNA pooling in predicting differences in allele frequency, we individually genotyped 12 random SNPs in all the participants. This includes 11 SNPs that did not reach the predefined threshold in the pooled association stage and one SNP, rs7785891, which did reach the threshold. Pearson’s correlation coefficient between the mean RAS scores and the allele frequency was calculated at r = 0.65. This correlation is considerably higher than another study that used pooled DNA obtained from cheek swabs on the same platform, though lower than the correlation reported for DNA obtained from blood samples [24].

## Results

**Fig 1:**
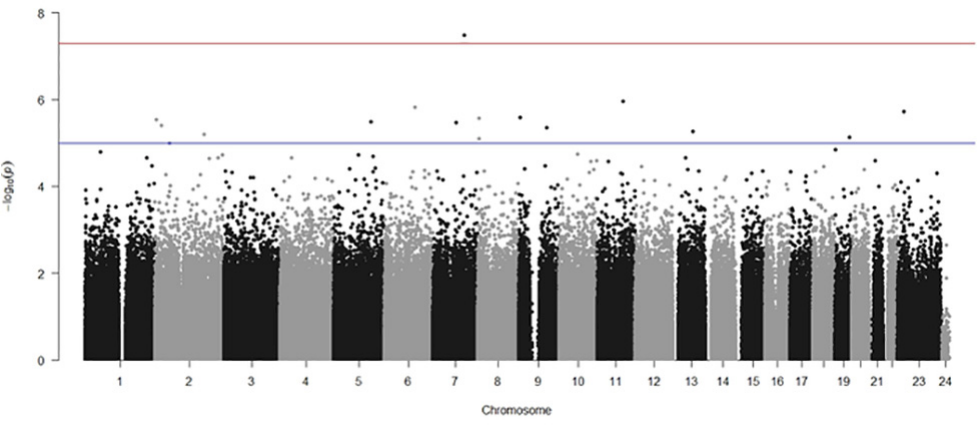
Manhattan plot of the SNPs tested in the pooled DNA association stage.

**Fig 2:**
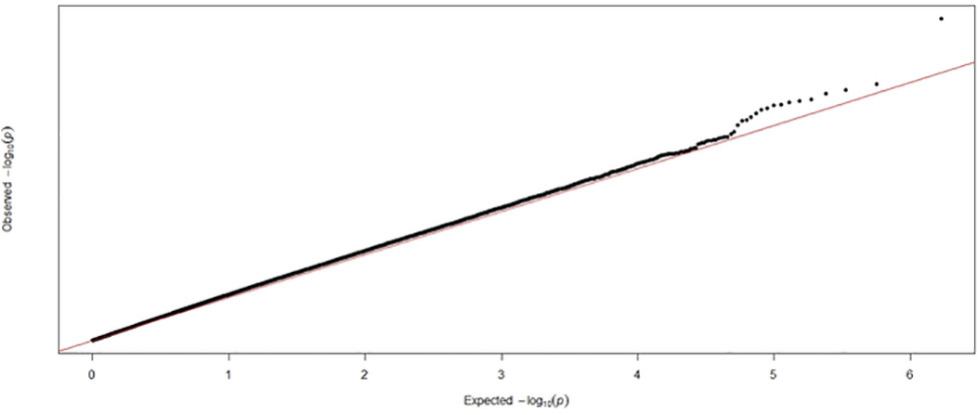
Quantile-quantile plot of the SNPs tested in the pooled DNA association stage.

In the DNA-pooling stage, 11 SNPS passed the threshold of significance and quality control. Additionally, 5 SNPs with p-values below 1 x 10^−5^ failed quality control at the pooling stage (Fig 1). All the 11 SNPs were individually genotyped and all the SNPs passed quality control in the individual genotyping phase. Three SNPs were nominally significant at p <0.05 in this stage (rs7785891, rs1268055, and rs2782448). None of the SNPs survived correction for multiple testing using Bonferroni correction. None of these three nominally significant SNPs from the individual genotyping stage were significant in the PGC ASC dataset. Results are summarised in Table 1. A Q-Q plot of the results from the pooling stage is provided in Fig 2.

**Table 1:** Summary of results.

RSID	Chr	Closest Gene	P-value (pooled genotyping)	P-value (individual genotyping)	Allele 1 (minor allele)	Freq of allele 1	Allele 2 (major allele)	Freq of allele 2	Chi-Sq	Odds Ratio	P-value (PGC cohort)
rs2782448	13	*KLHL1*	5.32759E-06	0.005566	G	0.2872	C	0.7128	7.686	0.7075	0.4867
rs1268055	6	*ARMC2*	1.49962E-06	0.03515	G	0.4468	A	0.5532	4.438	0.782	0.3917
rs7785891	7	*ZNF277/DOCK4*	3.27832E-08	0.04701	G	0.4094	C	0.5906	3.945	1.268	0.4532
rs25870	5	*FSTL4*	3.23677E-06	0.09446	C	0.2555	T	0.7445	2.797	0.8021	NA
rs7826102	8	*CSMD1 /LOC100287015*	2.73904E-06	0.1654	G	0.09464	C	0.90536	1.924	0.7685	NA
rs4665507	2	*LOC645949 / KLHL29*	4.00377E-06	0.5449	G	0.1098	A	0.8902	0.3665	0.8959	NA
rs7047415	9	*PTCH1 /LINC00476*	4.41161E-06	0.576	T	0.1036	C	0.8964	0.3127	1.116	NA
rs11901152	2	*NRXN1 / ASB3 /*	9.99301E-06	0.4843	A	0.2927	G	0.7073	0.4892	0.9155	NA
rs1036557	8	*CSMD1*	7.96515E-06	0.5411	G	0.4173	C	0.5827	0.3735	0.9298	NA
rs13005010	2	*ITGA6*	6.31853E-06	0.2523	A	0.113	G	0.887	1.31	1.249	NA
rs1526483	7	*SEMA3A*	3.40181E-06	0.908	G	0.1232	A	0.8768	0.01336	0.9796	NA

## Discussion

The current study used pooled DNA analysis to identify common variants associated with AS. Using pooled DNA we scanned the genome for SNPs that had a difference in allele frequencies between the case groups and the control groups. A threshold of 1 x 10^−5^ was selected in the pooling stage due to the loss of power during DNA pooling and to control for false negatives. SNPs which had p-values below the pre-defined threshold were treated as candidate SNPs and genotyped individually in the same group of individuals to more accurately estimate allele frequencies. Of the 11 SNPs that crossed the threshold of significance in the pooling stage, only three remained nominally significant after the individual genotyping stage. These three SNPs were not significantly associated with ASC in a larger, more heterogeneous ASC cohort from the PGC consortium.

rs778589, the top performing SNP at the pooling stage, is an intronic SNP in *DOCK4*, a gene previously associated with ASC [25, 26]. rs2782448 is an intergenic SNP at 13q21. It is 371 kb from *KLHL1* and 7.5 kb from the 3’ end of RP11- 459J23.1, a LincRNA identified by the Gencode project. 13q21 has been previously implicated in both autism [27, 28] and Specific Language Impairment [29]. The third nominally significant SNP, rs1268055, is an intronic SNP in *ARMC2*, a gene with uncertain function in humans.

The major limitation of this study is power. First, DNA pooling retains only 68% percent of the power [19]. Second, even after only selecting for individuals with AS, no SNP remained significant after Bonferroni correction. This indicates that larger sample sizes are required to detect causative alleles of small effect sizes. There is a considerably high correlation between the two stages of analysis, yet of the eleven SNPs selected for individual genotyping, only three remained nominally significant at this stage. The top two associated SNPs that passed quality control in the pooled DNA analysis stage were both nominally significant at the individual genotyping stage. However, they did not remain significant after correction for multiple comparisons. Of the three nominally significant alleles, rs7785891 has an odds ratio above 1, whereas rs1268055 and rs2782448 have odds ratio below 1 (see Table 1). Finally, while all our participants reported Caucasian ancestry for at least two generations, population stratification can confound the results and lead to false positives [30]. There are currently no known methods to correct for population stratification for pooled DNA association studies, taking into account the polygenicity of the condition.

While the current study tested for association with AS, we also checked to see if the three nominally significant SNPs were significant in an ASC cohort. The direction of effect for all three SNPs was similar to the effect direction in our sample. However, none of the three SNPs were nominally significant in the PGC ASC cohort. This may be due to a) the heterogeneity of the PGC cohort compared to our study cohort, since the former did not stratify for language delay, and/or b) the design of the association study being different (a family based association study based on trios vs a population based study), which may lead to different signal-to-noise ratios. However, it needs to be highlighted that the effect sizes for the SNPs in both the samples were small. This underscores the need for larger sample sizes to effectively identify common variants.

In conclusion, we report the identification three SNPs (rs1268055, rs7785891 and rs2782448) as nominally associated with AS using a genome-wide pooled DNA association study. rs2782448 and rs1268055 lie in genetic loci previously implicated in ASC. None of the SNPs remained significant after Bonferroni correction, underscoring the need for larger sample sizes to uncover alleles with small effect sizes. This is the first genome-wide case-control association study to test common variants for association with AS.
